# Economic Uncertainties in Retirement: Risk Factors, Strategies, and Resilience

**DOI:** 10.1093/geroni/igaa057.2392

**Published:** 2020-12-16

**Authors:** Christine Bishop, Karen Zurlo

## Abstract

Even with forethought and planning, a lot can threaten economic wellbeing in the years ahead for older adults retiring at typical retirement ages. Although results for any individual cannot be predicted with certainty, some risks are quantifiable: for example, mortality/ longevity and disability risks are reasonably well-defined. Risk of dementia is not so well understood, and may be changing. Financial risk might be seen as manageable, but older adults relying on retirement income sources can be especially vulnerable to unprecedented shocks to the general economy. We consider four aspects of this dilemma. First, older adults retiring with outstanding debts may have difficulty weathering financial shocks. Our first presentation provides up-to-date information about trends in indebtedness at older ages, especially focusing on newly salient types of indebtedness: medical and student loan debt, and debt incurred to smooth finances in the recent recession. Stewardship of finances during retirement can be a challenging personal management undertaking. Our second presentation will consider how dementia can complicate this process. Protection against outliving one’s resources is more complex and costlier in the era of defined contribution retirement accounts. Our third presentation will discuss strategies to combine retirement assets, including Social Security claiming, to hedge longevity risk. Finally, needs for long-term services and supports may be met with either paid or informal (family) care, or both, but cannot be predicted with certainty. Our fourth presentation examines the long-term impacts on families due to the difficulty in insuring against this risk. Economics of Aging Interest Group Sponsored Symposium.

